# The Safety of Abiraterone Acetate in Patients with Metastatic Castration-Resistant Prostate Cancer: An Individual-Participant Data Meta-Analysis Based on 14 Randomized Clinical Trials

**DOI:** 10.3390/cancers17172747

**Published:** 2025-08-23

**Authors:** Amy L. Shaver, Nikita Nikita, Swapnil Sharma, Scott W. Keith, Kevin K. Zarrabi, Wm. Kevin Kelly, Grace Lu-Yao

**Affiliations:** 1 Department of Medical Oncology, Sidney Kimmel Medical College, Thomas Jefferson University, Philadelphia, PA 19107, USAswapnil.sharma@jefferson.edu (S.S.);; 2 Sidney Kimmel Comprehensive Cancer Center, Thomas Jefferson University, Philadelphia, PA 19107, USA; 3 Division of Biostatistics and Bioinformatics, Department of Pharmacology, Physiology, and Cancer Biology, Sidney Kimmel Medical College, Thomas Jefferson University, Philadelphia, PA 19107, USA; 4 College of Population Health, Thomas Jefferson University, Philadelphia, PA 19107, USA

**Keywords:** serious adverse events, metastatic castration-resistant prostate cancer, fracture, neuromuscular toxicity

## Abstract

**Background/objectives**: Multiple systemic treatments are available for metastatic castration-resistant prostate cancer (mCRPC), with unclear safety profiles. This study seeks to describe the safety determined in randomized clinical trials of a systemic treatment for mCRPC and whether safety differs by age. **Methods**: We utilized individual patient data from industry-funded phase 2/3 trials in mCRPC on abiraterone acetate (AA). Vivli, a clinical trial repository site, was used. One investigator independently performed screening. Relative effects of treatment were assessed with frequencies and odds of serious adverse events (SAEs). The Preferred Reporting Items for Systematic Reviews and Meta-analyses guideline was used. Subgroup analysis measured odds of SAEs as modified by age. **Results**: We identified 14 trials with 4296 patients. The median age of participants was 69 years. Nearly all participants experienced at least one adverse event (98.4% abiraterone, 97.3% standard of care [SOC]). More serious adverse events (grade 3 or 4) and deaths (grade 5) occurred in those receiving SOC (71.8%) compared to abiraterone (64.1%). The most frequent adverse event category was “Musculoskeletal and Connective Tissue Disorders”. The most frequent event types included anemia, back pain, hypertension, fatigue, hypokalemia, and bone pain. The odds of all events were lower in those receiving abiraterone compared to SOC. Odds of a serious musculoskeletal event were lower in older subjects by 22% (OR 0.78; 95% CI 0.63, 0.96). **Conclusions**: In this IPD meta-analysis, abiraterone acetate provides no greater risk of SAE in those receiving abiraterone than those receiving SOCs. Patients in the RCTs are younger and healthier than those in the general population; consequently, the results of RCTS might not be applied to the general population, especially those under-represented in the RCTs. There is a need to further evaluate abiraterone-related fractures and neuromuscular toxicities (NMTs) as key outcomes to gain insight into risk factors related to these adverse events. A real-world prospective study is warranted to examine the overall risks and benefits associated with treatment.

## Simple Summary

Abiraterone acetate is one of the most common drugs used in the treatment of metastatic prostate cancer. As with many therapies, adverse events can occur during treatment. Some of these events can be serious enough to require hospitalization and a pause in or the discontinuation of treatment. This study was conducted in order to determine the most common serious adverse events that occur in patients with metastatic castration-resistant prostate cancer. We analyzed individual patient data from clinical trials in a meta-analysis. We found that musculoskeletal and connective tissue disorders were the most common serious adverse events.

## 1. Introduction

Both androgen deprivation therapy (ADT) and androgen receptor pathway inhibitor (ARPI) treatments in patients with prostate cancer (PCa) have been linked to negative musculoskeletal outcomes [1,2]. Androgens play an important role in maintaining muscle mass, strength, and neuromuscular function [3,4]. Studies show that treatment with Abiraterone, an ARPI and ADT treatment, results in a rapid and significant decline in circulating androgens [5], which in turn disrupts the process of bone remodeling, ultimately leading to overall bone loss and osteoporosis [6,7]. Additionally, the loss of androgens contributes to muscle atrophy, weakness, and neuromuscular dysfunction, which can further predispose patients to falls and fractures [3]. Abiraterone and ADT use have also been associated with a significant increase in adiposity (both subcutaneous and visceral) and reduced muscle mass (sarcopenia) [8,9], increasing the risk of falls and fractures [7,10]. Abiraterone is often given in combination with prednisone, and the long-term use of prednisone is also known to increase the risk of osteoporosis and fractures [11,12].

ADT and Abiraterone treatments are also often associated with neuromuscular toxicities that can impair muscle function, exacerbate frailty, and increase the risk of falls [13,14,15]. Decreased testosterone levels due to Abiraterone and ADT lead to muscle atrophy, reduced muscle strength, and increased fatigue, contributing to an overall decline in physical function [16], or general musculoskeletal discomfort, limiting mobility and daily activities. Abiraterone treatment can also cause secondary mineralocorticoid excess, which can lead to electrolyte imbalances such as low potassium levels, contributing to muscle cramps, weakness, and even neuromuscular dysfunction [12,15,17,18,19].

Most patients with PCa are over 65 years at presentation [20]. However, the median age of patients with PCa participating in clinical trials is 6.5 years younger than the age at diagnosis in the general population [21]. This can present an issue for clinicians treating older patients due to a lack of guidelines or evidence based on clinical trial data involving older adults [22].

This study examined whether older age influences the incidence of adverse events. This information can significantly improve treatment decision-making and have a significant impact on patient outcomes. Evaluating the risk of fractures and NMTs linked to abiraterone treatment will also help to reduce the burden of these adverse effects and enhance overall treatment outcomes for PCa patients.

## 2. Materials and Methods

**Figure 1:**
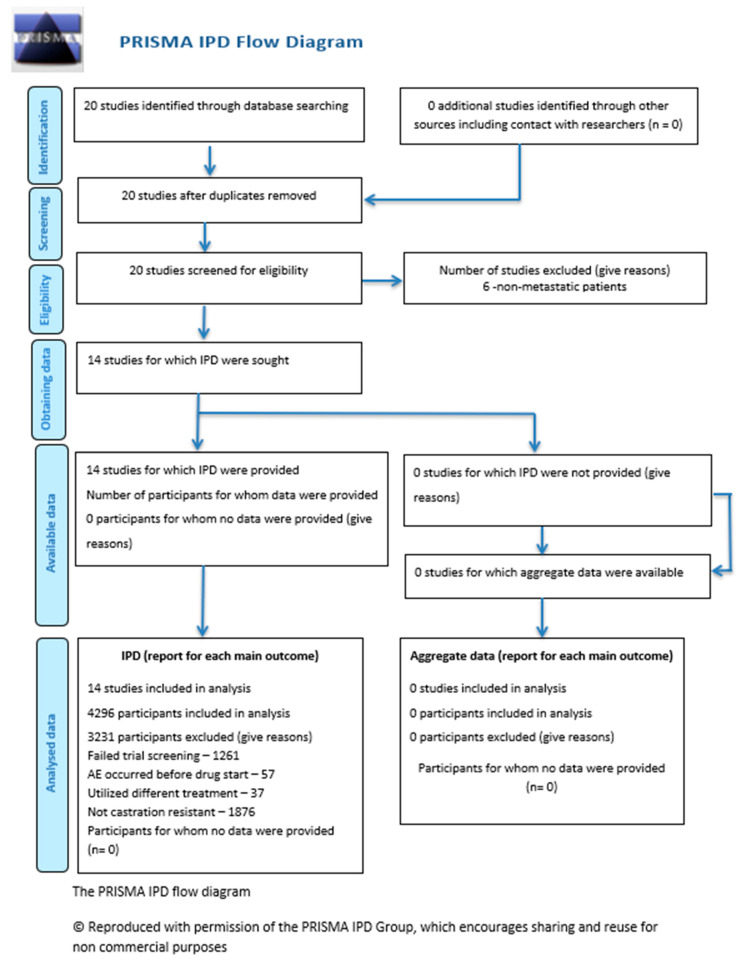
The PRISMA IPD flow diagram.

This individual participant data meta-analysis (IPD MA) was conducted within the Vivli safe haven/trusted research environment (hereafter, Vivli). The current report presents findings from the assessment of age as a potential modifier of treatment effect. Findings are reported according to the Preferred Reporting Items for Systematic Reviews and Meta-analyses (PRISMA) reporting guidelines (Figure 1 and Table S1). The protocol for these analyses can be found on both the Yale University Open Data Access (YODA) and Vivli data repository sites.

**Figure 2:**
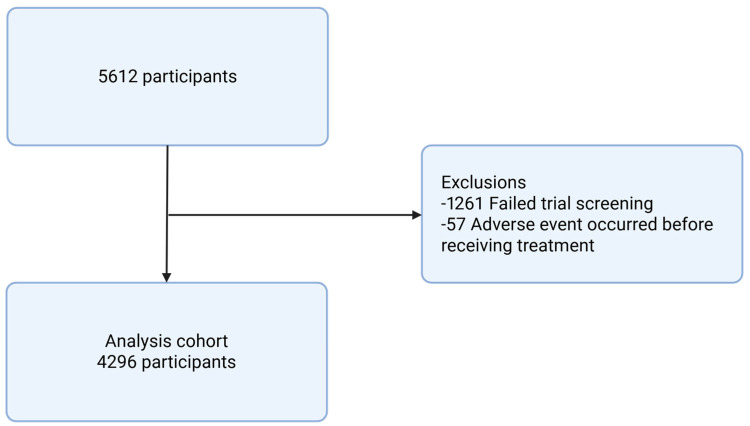
Consort diagram.

Eligibility Criteria and Search Strategy: Eligible studies were randomized clinical trials with available IPD that enrolled adults aged 18 years or older diagnosed with metastatic castration-resistant prostate cancer, assessing the efficacy of abiraterone acetate. The comparator arm, when it existed, in this study referred to as standard of care (SOC), was eligible so long as the study evaluated outcomes of abiraterone, examples include both a genuine placebo and chemotherapy. Participants needed to pass screening, and adverse events had to occur after the first day of the trial to be considered for analysis (Figure 2).

Data Extraction: Drug names and age at baseline were extracted and summarized from the trial data. The Common Terminology Criteria for Adverse Events (CTCAE) category, grade, and specific types of adverse events were extracted. The definitions of neuromuscular toxicities and fractures were harmonized across trials using adjudicated events. Individual-level trial data were cleaned and harmonized in the Vivli safe haven/trusted research environment (hereafter, Vivli). Data on adverse events were also extracted from the trials, focusing on serious adverse events (grades 3, 4, and 5). The frequency and odds ratio of adverse events were identified and examined.

Statistical Analysis: All analyses, as they required access to IPD, were conducted within Vivli. This approach was chosen to meet the terms of the data sharing agreement and to maximize reuse and ensure reproducibility. The age distributions were summarized using IPD. A two-stage meta-analysis approach was utilized due to the large number (n) of participants (n = 4296) [23]. In the first stage, unadjusted logistic regression models were used to determine the odds of adverse events of interest (any serious adverse event, and musculoskeletal, anemia, back pain, hypertension, fatigue, and neuromuscular toxicity, and fracture events) for each individual study, and models were summarized using 95% confidence intervals and standard errors. The second stage utilized the R-package metafor [24]. The second stage of analysis utilized a random-effects model where weighting considered sampling variance as well as the estimated heterogeneity between studies [25,26]. For all models, SOC was the reference treatment. *p*-values are only reported for the pooled effect size. Models were summarized using 95% confidence intervals (CI) for the main effect, which represents the plausible range of the effect. As a safety analysis, the presentation of *p*-values is not generally recommended [27]. None of the analyses used formal adjustment for multiple testing, in line with Rothman’s argument and with an eye to safety, we chose to err on the side of caution and not miss a potentially important finding related to patient harm [28]. All analyses were conducted using R version 4.4 (R Foundation for Statistical Computing). Generative artificial intelligence (GenAI) was not used in either the analysis or reporting of this study.

## 3. Results

The 14 studies used for analysis are listed in Table 1; each trial was restricted to men with mCRPC and evaluated the efficacy and/or effectiveness of abiraterone. Participants were followed and adverse events were recorded from study start up to 5 years; the overall length of study follow-up ranged from 365 to 1800 days. Participant characteristics according to treatment appear in Table 2. The median age of participants was 69 years, with a range from 39 to 97 years old. Nearly all participants experienced at least one adverse event of any grade (98.4% abiraterone, 97.3% SOC) (Table 2). More serious adverse events (grade 3 or 4) and deaths (grade 5) occurred in those receiving SOC (71.8%) compared to abiraterone (64.1%).

The top 5 CTCAE categories and specific adverse event types are summarized in Table 3. The most frequent CTCAE category was “Musculoskeletal and Connective Tissue Disorders,” with 780 events occurring in subjects receiving abiraterone and 369 in subjects receiving SOC. The most frequent event types included anemia, back pain, hypertension, and fatigue. Anemia, back pain, hypertension, and fatigue were each in the top 4 events for either abiraterone or SOC.

**Figure 3:**
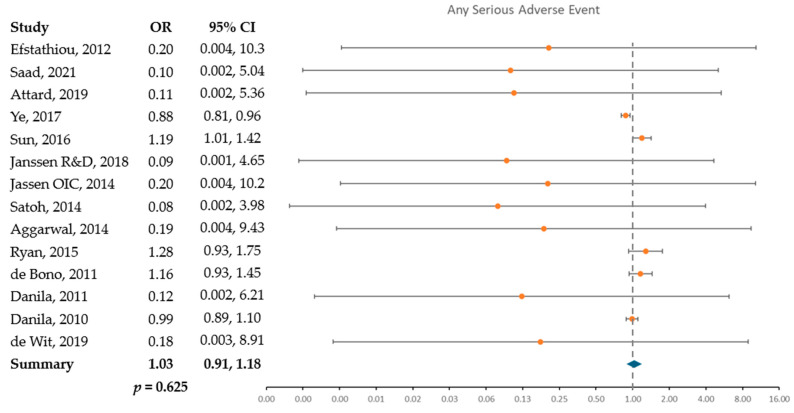
Odds of serious adverse events in abiraterone subjects compared to SOC based on 14 included studies [18,29,30,31,32,33,34,35,36,37,38,39,40,41]. Odds ratio compares odds of an event in those receiving abiraterone to odds of an event in those receiving SOC. Standard of care (SOC) refers to patients and events who come from trials with a comparator arm and includes SOC, placebo, and other anti-neoplastic agents. Above 1 indicates an event occurring in abiraterone participants, below 1 indicates an event occurring in SOC participants.

Odds of serious adverse events, both for individual and pooled studies, in those receiving abiraterone compared to SOC are presented in Figure 3. The odds of all events were lower in those receiving abiraterone compared to SOC. Effect sizes ranged from a 99% reduction in fatigue (odds ratio [OR] 0.01; 95% confidence interval [CI] 0.002, 0.07) to 89% lower odds for musculoskeletal complaints (OR 0.11; 95% CI 0.03, 0.397), as shown in Figure S1. All results were statistically significant except for the odds of any serious adverse event (OR 1.03; 95% CI 0.91, 1.18).

**Figure 4:**
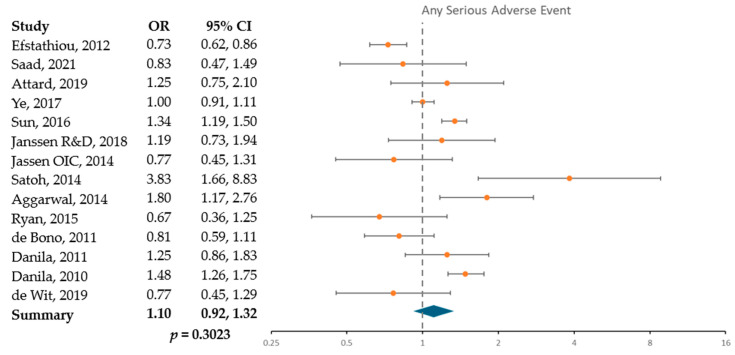
Odds of serious events in older compared to younger abiraterone subjects based on 14 included studies [18,29,30,31,32,33,34,35,36,37,38,39,40,41]. Odds ratio compares the odds of an event in older subjects (≥70) receiving abiraterone to the odds of an event in younger subjects (<70) receiving abiraterone. Above 1 indicates an event occurring in older people, below 1 indicates an event occurring in younger people.

Figure 4 presents the odds of serious adverse events in those receiving abiraterone, comparing older subjects (70 years or older) to younger subjects (under 70 years of age). Odds of a serious musculoskeletal event were lower in older subjects by 22% (OR 0.78; 95% CI 0.63, 0.96), as shown in Figure S2. No other events reached the level of statistical significance.

## 4. Discussion

Our meta-analysis of 14 randomized clinical trials (RCTs) aimed to investigate the safety of abiraterone acetate in patients with mCRPC and to determine if event odds differed according to age. To the best of our knowledge, this study was the first to investigate these elements according to the age of the participant using an IPD meta-analysis format. While the results showed no significant increase in SAE according to age, the lower risk of musculoskeletal events in the older age group compared to the younger group suggested that only the fittest older patients were included in the RCTs. Consequently, the results of the RCTs might not be applicable to the general population and real-world study on older patients are needed. The data of the 14 RCTs included in the IPD MA provided accurate estimates. All included RCTs [15,18,29,30,31,33,34,35,36,37,38,39,40,41] confirmed that abiraterone acetate achieved a significant benefit compared to SOC according to the development of serious adverse events in patients with mCRPC. However, no relevant, high-quality RCTs have been conducted to directly compare the efficacy of abiraterone acetate between younger and older patients with mCRPC. It was meaningful for us to identify whether abiraterone acetate achieved a different effect according to age in mCRPC by subgroup analysis.

The current study found that more serious adverse events occurred more frequently in the SOC group (71.8%) than in the abiraterone acetate group (64.1%). Odds of serious adverse events were lower in abiraterone acetate users than in SOC participants. In subgroup analysis, young patients with mCRPC had 22% higher odds of musculoskeletal and connective tissue disorder than older patients. It may be that younger patients had a more aggressive disease, or similar to Satoh et al., the positive PSA response found in patients both over 65 and over 75 was more robust than that found in younger patients, potentially leading to lower skeletal muscle events [34]. Interestingly, Williams et al. found no outstanding differences in treatment response between patients with mCRPC under 55 and those 55 years and older [42]. This finding warrants additional investigation to determine if this is a dose effect or a differential effect according to age.

We observed a higher incidence of overall adverse events with the use of abiraterone acetate (98.4%) compared to (97.3%), though given the distribution of serious adverse events, this finding involves mainly grade 1–2 adverse events, a finding similar to a recent study [43]. It is important to note that grade 1 and 2 events do not often result in drug disruption or discontinuation. The most common adverse event was anemia in both the abiraterone acetate and SOC groups. Other common adverse events are in line with previously published studies, namely, back pain [44], hypertension [45], and fatigue [46]. In all cases, the effect was less in the abiraterone group than in the SOC group. A recent IPD meta-analysis to determine who benefits more from an androgen receptor pathway inhibitor (ARPI) vs. docetaxel plus ADT doublet (conducted by the Systemic Treatment Options for Cancer of the Prostate [STOPCAP] Collaboration) in patients with metastatic hormone-sensitive prostate cancer found the relative benefit of ARPIs on progression free survival (PFS) increased with younger age (interaction *p* = 0.034); further, effects were similar for overall survival (OS, age interaction *p* = 0.035) [47]. STOPCAP found that the effect of age was most pronounced in the abiraterone trials [47]. This is increasingly important, as advances in oncologic care have lengthened the lives of men with prostate cancer [48]. As discussed by PCa expert Dr. N. Agarwal, safety of patients with mCRPC, who are living longer than ever before, depends on prior therapies and disease status, it is imperative of oncologists to carefully monitor their patients and take the patient’s current physiological state, including comorbidity, into consideration as part of that monitoring [49]. Furthermore, it is critical to further evaluate the effect of the loss of muscle mass that occurs in men treated with ADT and newer therapies [50]. Lean muscle can be lost after only a short duration of ADT [51], measurement of lean mass is not a parameter commonly assessed in clinical trials or in general practice and a thorough evaluation of muscle quality is warranted among mCRPC patients to see if outcomes are similar to those found in other cancers [52,53,54].

This IPD meta-analysis has several strengths, including the use of individual-level patient data and the inclusion of 14 RCTs. The study also suffers from some limitations due to heterogeneity in the RCT’s study designs including differences in standard of care arm, while adjustment was made for study differences in the aggregated random effects model, a residual effect is possible. This is most apparent as a differing effect seen in three of the studies [32,37,38], two of which involve chemotherapy-naïve participants and the third which studied men post-docetaxel failure. The variation in studies included both randomized, double-blind trials, and open-label trials. This may account for the finding of this study of a lack of hypertension risk which is contrary to published reports with more homogenous data [45]. However, the combination of heterogenous studies can enhance precision and help identify small but potentially meaningful effects that may have been missed in a sampling of non-heterogenous studies. Safety analyses should be interested in signal detection of adverse events which are then explored in real-world, prospectively collected data [55,56]. Future work should include subset analysis based on patient and study variations. This study did not collect information on prior treatment, concurrent medications, or time to adverse events, all of which could have offered insight into the findings and their interpretation. This study did not include adjustment for baseline Karnofsky and ECOG values which could have influenced the results found between older and younger participants. However, the older participants found in a clinical trial tend to be healthier than their real-world counterparts, who are often excluded from clinical trials due to their comorbidity burden [57]. It is important to note that while recording adverse events is critical to drug development, multiple studies have found that event reporting can be varied between clinical trials [58,59] and underreporting of adverse events among older cancer patients in trials has been reported [60]. Finally, Arana-Chicas et al. have reported that CTCAE does not fully capture events as reported by older patients [61]. This may limit the generalizability of the findings of this study to the general population who would not necessarily be eligible to participate in a clinical trial. While all men included had mCRPC and received abiraterone, some were post-docetaxel [30,34,36,37] and some were chemotherapy naïve [32]. While most of the studies were Phase II/III, Aggarwal presented results of a Phase I/II dose escalation trial [33]. The studies included did not release information on comorbidity, and so a comparison based on pre-existing conditions (along with age) was not presented. Future studies should focus on a more uniform treatment population.

## 5. Conclusions

While the risk of adverse events following abiraterone acetate for patients with metastatic castration-resistant prostate cancer is not higher than that for those receiving SOCs, the lower median age in the RCT compared with that of the general population and the lower risk of musculoskeletal disorders in the older age group suggests the selection of relatively fit older patients in RCTs impacted both the outcomes of this study and generalizability. It is crucial that future investigation gathers high-quality real-world data and collect prospective data on musculoskeletal parameters and conditions to identify factors that could impart greater risk for serious adverse events for patients who are often under-represented in RCTs due to pre-existing comorbidities.

## Figures and Tables

**Table 1:** Studies included in the analysis.

National Clinical Trial (NCT)	Author, Year	Study	Number (n) of Included Subjects
NCT00544440	Efstathiou, 2012	An Observational Study of Continuous Oral Dosing of an Irreversible CYP17 Inhibitor, Abiraterone Acetate (CB7630), in Castration-Resistant Prostate Cancer Patients Evaluating Androgens and Steroid Metabolites in Bone Marrow Plasma [29]	56
NCT00474383	Danila, 2010	A Phase II Open Label Study of CB7630 (Abiraterone Acetate) in Patients with Advanced Prostate Cancer Who Have Failed Androgen Deprivation and Docetaxel-Based Chemotherapy [30]	45
NCT00485303	Danila, 2011	A Phase II Open Label Study of CB7630 (Abiraterone Acetate) and Prednisone in Patients with Advanced Prostate Cancer Who Have Failed Androgen Deprivation and Docetaxel Based Chemotherapy [31]	57
NCT00638690	de Bono, 2011	A Phase 3, Randomized, Double-blind, Placebo-Controlled Study of Abiraterone Acetate (CB7630) Plus Prednisone in Patients with Metastatic Castration-Resistant Prostate Cancer Who Have Failed Docetaxel-Based Chemotherapy [18]	1073
NCT00887198	Ryan, 2015	A Phase 3, Randomized, Double-blind, Placebo-Controlled Study of Abiraterone Acetate (CB7630) Plus Prednisone in Asymptomatic or Mildly Symptomatic Patients with Metastatic Castration-Resistant Prostate Cancer [32]	995
NCT00473746	Aggarwal, 2014	A Phase I/II Open Label Dose Escalation Study of the 17α-Hydroxylase/C17,20-lyase Inhibitor, Abiraterone Acetate in Hormone Refractory Prostate Cancer [33]	65
NCT01795703	Satoh, 2014	A Phase II Study of JNJ-212082 (Abiraterone Acetate) in Metastatic Castration-Resistant Prostate Cancer Patients Who Have Received Docetaxel-based Chemotherapy [34]	43
NCT01424930	Jassen OIC, 2014	An Open-Label Study to Determine the Short-Term Safety of Continuous Dosing of Abiraterone Acetate and Prednisone in Modified Fasting and Fed States to Subjects With Metastatic Castration-Resistant Prostate Cancer [35]	23
NCT01685983	Janssen R&D, 2018	A Phase 2 Open Label Study of Abiraterone Acetate (JNJ-212082) and Prednisolone in Patients with Advanced Prostate Cancer Who Have Failed Androgen Deprivation and Docetaxel-Based Chemotherapy [36]	80
NCT01695135	Sun, 2016	A Phase 3, Randomized, Double-blind, Placebo-Controlled Study of Abiraterone Acetate (JNJ-212082) Plus Prednisone in Patients with Metastatic Castration-Resistant Prostate Cancer Who Have Failed Docetaxel-Based Chemotherapy [37]	213
NCT01591122	Ye, 2017	A Phase 3, Randomized, Double-Blind, Placebo-Controlled Study of Abiraterone Acetate (JNJ-212082) Plus Prednisone in Asymptomatic or Mildly Symptomatic Patients with Metastatic Castration-Resistant Prostate Cancer [38]	309
NCT01867710	Attard, 2019	A Randomized Phase 2 Study Evaluating Abiraterone Acetate With Different Steroid Regimens for Preventing Symptoms Associated With Mineralocorticoid Excess in Asymptomatic, Chemotherapy-Naïve and Metastatic Castration-Resistant Prostate Cancer (mCRPC) Patients [39]	162
NCT02257736	Saad, 2021	A Phase 3 Randomized, Placebo-controlled Double-blind Study of JNJ-56021927 in Combination with Abiraterone Acetate and Prednisone Versus Abiraterone Acetate and Prednisone in Subjects with Chemotherapy-naïve Metastatic Castration-resistant Prostate Cancer (mCRPC) [40]	938
NCT02485691	de Wit, 2019	A Randomized, Open Label, Multicenter Study of Cabazitaxel Versus an Androgen Receptor (AR)-Targeted Agent (Abiraterone or Enzalutamide) in mCRPC Patients Previously Treated With Docetaxel and Who Rapidly Failed a Prior AR-targeted Agent (CARD) [41]	237

Abbreviations: n, number; NCT, National Clinical Trial.

**Table 2:** Characteristics and serious adverse event summary of trial participants *.

Characteristic	Abiraterone (n = 3143)	Standard of Care ^ (n = 1153)	Overall (n = 4296)
Age			
Mean (SD)	68.9 (9.10)	69.1 (8.80)	69.0 (9.02)
Median (range)	69.0 (39.0, 97.0)	70.0 (39.0, 97.0)	69.0 (39.0, 97.0)
With serious adverse events (Grade 3–5)	2014 (64.1)	828 (71.8)	2842 (66.2)

* Data is presented as mean (standard deviation), median (range), or number (n [%]); every subject is counted a single time for each applicable row and column. ^ Standard of care (SOC) refers to patients and events who come from trials with a comparator arm and includes SOC, placebo, and other anti-neoplastic agents.

**Table 3:** Top 4 CTCAE categories and types of serious adverse events ^.

CTCAE Category	Abiraterone n = 2412 *	SOC n = 1002 *
Musculoskeletal and Connective Tissue Disorders	780	369
Metabolism and Nutrition Disorders	643	210
Investigations	545	257
General Disorders and Administration Site Conditions	444	166
**Serious Event Type**	**Abiraterone** n = 928 *	**SOC** n = 329 *
Anemia	258	96
Hypertension	250	79
Back Pain	211	86
Fatigue	209	68

* Number (n) of events occurring in users of each treatment, either abiraterone or SOC. ^ Standard of care (SOC) refers to patients ad events who come from trials with a comparator arm and includes SOC, placebo, and other anti-neoplastic agents.

## Data Availability

The data used for this study may be attained through successful application to the Vivli Data repository and agreement by the data contributors.

## Supplementary Materials

The following supporting information can be downloaded at: https://www.mdpi.com/article/10.3390/cancers17172747/s1, Table S1: PRISMA-IPD Checklist; Figure S1: Odds of events in total cohort; Figure S2: Odds of events comparing older to younger abiraterone users.

## Abbreviations

The following abbreviations are used in this manuscript:

AA	Abiraterone Acetate
ADT	Androgen Deprivation Therapy
ARPI	Androgen Receptor Pathway Inhibitor
CI	Confidence Interval
CTCAE	Common Terminology Criteria for Adverse Events
DOD	Department of Defense
Inc	Incorporated
IPD	Individual Patient Data
IPD-MA	Individual Patient Data-Meta-Analyses
LLC	Limited Liability Company
MA	Meta-analysis
mCRPC	Metastatic Castration-Resistant Prostate Cancer
NCI	National Cancer Institute
NIH	National Institutes of Health
NMT	Neuromuscular Toxicity
OR	Odds Ratio
OS	Overall Survival
PCa	Prostate Cancer
PFS	Progression Free Survival
PRISMA	Preferred Reporting Items for Systematic Reviews and Meta-Analyses
RCT	Randomized Clinical Trial
SAE	Serious Adverse Event
STOPCAP	Systemic Treatment Options for Cancer of the Prostate
YODA	Yale University Open Data Access
