# Supplementary figures and images for: The Safety of Abiraterone Acetate in Patients with Metastatic Castration-Resistant Prostate Cancer: An Individual-Participant Data Meta-Analysis Based on 14 Randomized Clinical Trials
Source: Cancers (Basel). 2025 Aug 23;17(17):2747. doi: 10.3390/cancers17172747 (PMC12427571; doi:10.3390/cancers17172747)

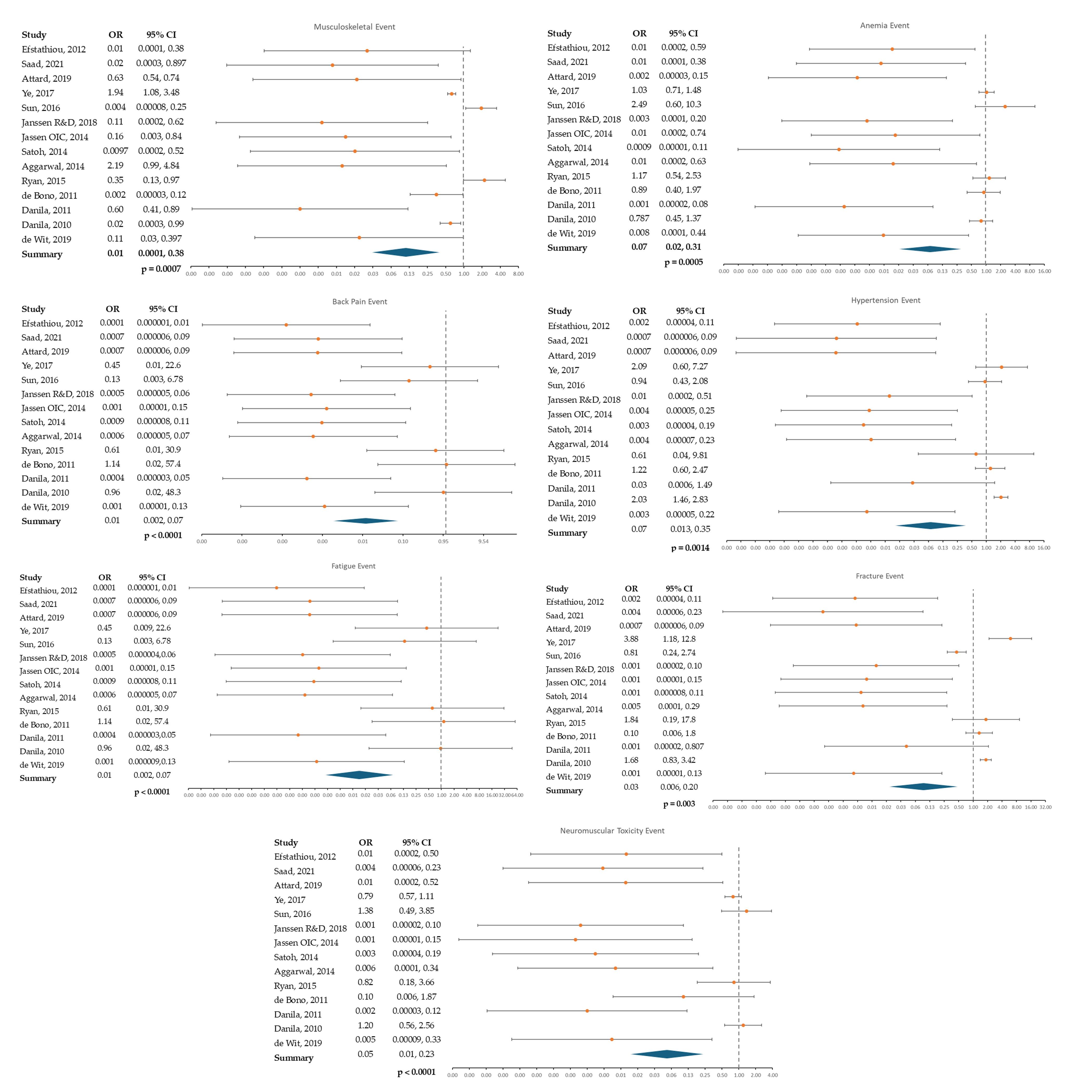

Supplement: Supplementary file 1 [file cancers-17-02747-s001.zip › Figure_S1.tif]

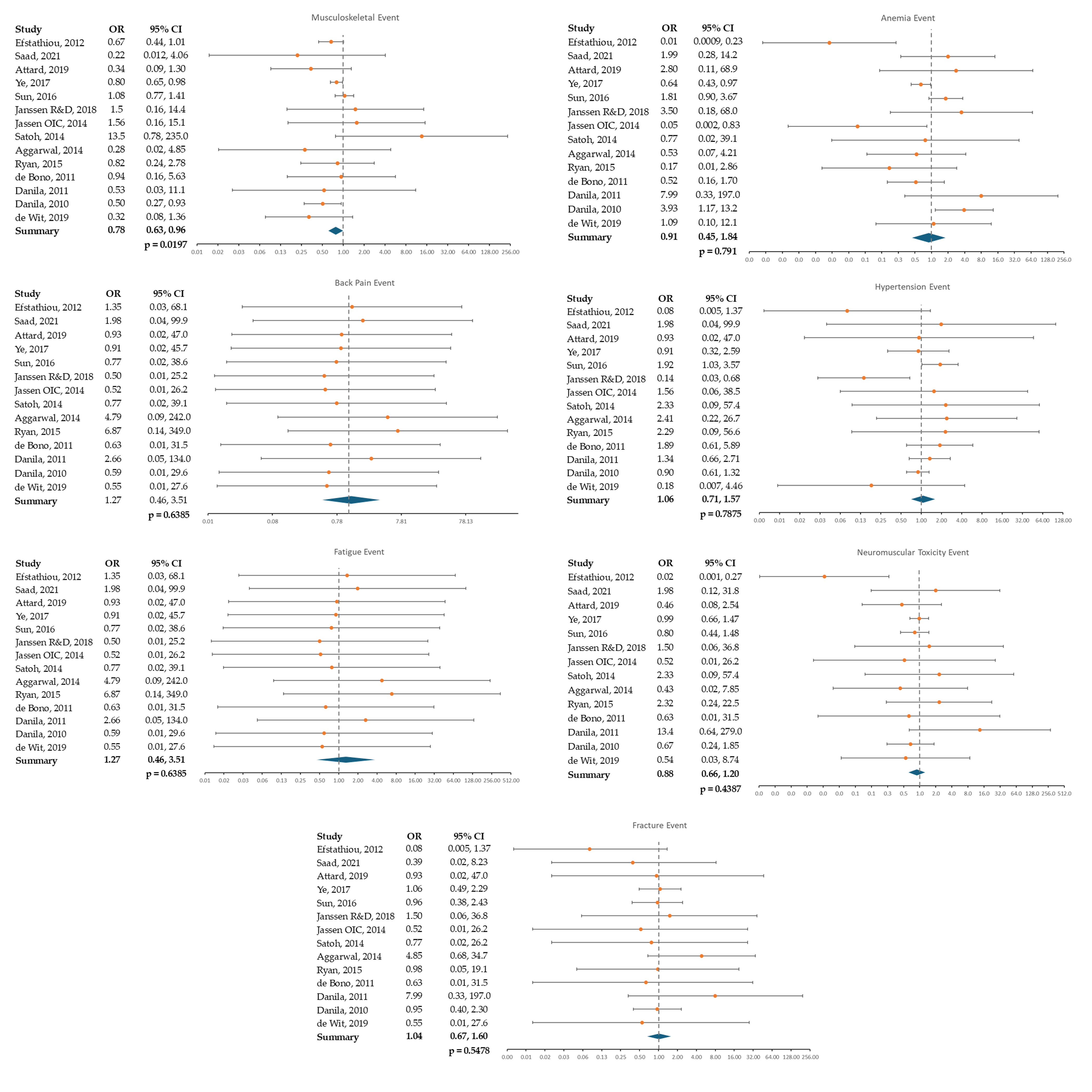

Supplement: Supplementary file 1 [file cancers-17-02747-s001.zip › Figure_S2.tif]
